# Training needs of investigators and research team members to improve inclusivity in clinical and translational research participation

**DOI:** 10.1017/cts.2020.554

**Published:** 2020-11-05

**Authors:** Susan R. Passmore, Dorothy Farrar Edwards, Christine A. Sorkness, Sarah Esmond, Allan R. Brasier

**Affiliations:** 1 Collaborative Center for Health Equity, School of Medicine and Public Health, University of Wisconsin–Madison, Madison, WI, USA; 2 Institute for Clinical and Translational Research, School of Medicine and Public Health, University of Wisconsin–Madison, Madison, WI, USA; 3 Department of Medicine, School of Medicine and Public Health, University of Wisconsin–Madison, Madison, WI, USA

**Keywords:** Recruitment, inclusivity, underserved populations, training, clinical research

## Abstract

Despite increasing attention to the importance of diverse research participants, success across the translational research spectrum remains limited. To assess investigator and research team training needs, we conducted a web-based survey exploring barriers in knowledge and practice. Respondents (*n* = 279) included those affiliated with the University of Wisconsin Institute for Clinical and Translational Research (ICTR). Although all respondents reported an abstract belief in the importance of diversity, factors associated with higher levels of best practices knowledge and implementation included: (1) use of federal funding; (2) having fewer years of experience; (3) recruiting healthy participants; and (4) having recruitment training.

## Introduction

Participants in health research are overwhelmingly White [1–3]. This remains true even when the condition under study is known to disproportionately impact underrepresented ethnic and racial minorities (URM) [1, 4]. The ongoing lack of diversity in biomedical research places unnecessary limitations on our knowledge of human variation and disease, the generalizability of research findings, and our ability to address health disparities [2, 3]. It also threatens the ethical principle of justice, which requires that the benefits of research are shared fairly [5]. For these reasons, the National Institutes of Health (NIH) has established guidelines and requirements to promote the inclusion of minorities in clinical research over the past three decades [6]. The US Food and Drug Administration has also established guidance to enhance diversity in industry-sponsored clinical trials [7]. Yet, recent studies have shown limited gains in diversity even while the number of clinical trials conducted globally has grown exponentially [2].

The persistence of this problem is also puzzling as the last 30 years have seen the emergence of a large and complex body of literature on the topic of URM recruitment. Participant distrust born of historical research abuse and continued discrimination in clinical encounters has been postulated as a primary cause [8, 9]. This work has, in turn, led to the development of a number of evidence-based, frequently community-engaged, approaches to promote the inclusion of URM in clinical research much of which has emerged from the Clinical and Translational Science Award (CTSA) Consortium members [10, 11]. These best practices in URM engagement include making use of community partners, community advisory boards, tailored recruitment materials, and the establishment of trust through long-term engagement. Yet, the lack of diversity in biomedical research remains [1–3].

In order to assess investigator training needs to support URM recruitment, we conducted a survey of researchers and team members affiliated with the UW-ICTR. Our goal was to explore: (1) attitudes and knowledge about the value of research participant diversity; (2) attitudes and knowledge about the URM best practices described in the literature; and (3) the use of URM recruitment best practices. Our intent was to use this needs assessment to directly inform the development of training programs at our CTSA.

## Materials and Methods

We fielded a web-based survey that consisted of items designed to: (1) identify “types” of participants based on role in research, type of research funding, years of experience in research, etc. and (2) determine knowledge, attitudes, and practices regarding participant recruitment with a specific focus on URMs. All materials and procedures were approved by the UW Madison Institutional Review Board (#2019–1211).

### Items

Items were developed with reference to existing instruments used in similar web-based surveys including the Building Trust Researcher Survey [12] and the University of South Carolina Clinical Trials Principal Investigators Survey [13]. Adapted questions from these surveys comprised of the conceptual items our instrument that covered knowledge, attitudes, and practices regarding URM engagement and best practices. Additional items regarding respondent characteristics (type of research, years in research) were selected from a previous internal UW ICTR evaluation survey of researchers on recruitment practices (not URM focused). The final instrument comprised of 23 items (some including subitems) including those meant to categorize the types of research conducted by respondents as well as to explore training experiences. Additional Likert-type items assessed attitudes and knowledge regarding URM recruitment and general populations “theoretically” (without reference to their own work) and these same attitudes and knowledge items with reference to participants’ specific research activities (e.g., “how much is recruiting racial and ethnic minority participants a priority in your research?”). Likert-scaled items were developed to reflect current best practices in URM recruitment such as community-engaged strategies (e.g., use of community advisory boards, community partnerships, diverse recruitment teams, tailored recruitment methods, etc.) [8]. Once compiled, the University of Wisconsin Survey Center (UWSC) provided an expert technical review of the instrument. Please see the supplementary materials for a copy of the survey.

### Survey Procedures

To ensure the impartiality of responses, the UWSC provided oversight of the survey administration which was conducted with Qualtrics survey software. Requests for participation were delivered via email including a personalized link. Three waves of reminder emails were sent to nonresponders. The survey was open for 4 weeks between February 24, and March 20, 2020.

### Respondents

Our sample consisted of UW Health and UW Madison current employees who were: (1) users of the UW Madison clinical research management system registered under the title of research coordinator, principal investigator, or co-investigator; (2) users of research support from the UW ICTR between 2017 and 2019; and (3) employees in the UW Madison School of Medicine and Public Health (SMPH) functioning in the role of research coordinator. Emails (1456) were distributed to current employees, which produced 313 participants who finished at least 90% of the survey (response rate of 21.5%). Thirty-four of these respondents were dropped from the analysis because they self-reported not engaging in human subjects research. Data from the remaining 279 respondents were included in the analyses.

### Data Analysis

As noted, respondents differed by type of research, research funding, research role, years of experience, and types of participants. Preliminary analyses consisted of comparisons of these groups on variables relative to URM recruitment attitudes, knowledge, and practices. To further these analyses, we constructed two composite scales regarding URM best practices derived from the literature (use of a community advisory board, partnership with community-based organizations, racial/ethnic concordance of research staff and participants, devoting extra time to recruit URM participants, and valuing diverse research participation) [8]. The developed scales were: (1) Best Practices knowledge (seven items; *α* .907) and (2) Best Practices implementation (five items; *α* .825). Basic descriptive statistics were computed for the demographic variables, individual survey items, and the two composite scales. Mann–Whitney *U* tests [14] were computed to determine if there were differences between two groups on our ordinal (Likert type)-dependent variables and *t*-tests for continuous variables.

## Results

### Sample Description

The majority of our respondents were affiliated with the SMPH (81.4%); were engaged in clinical research (72.4%); and recruited patients with a specific diagnosis or condition (72.8%) as opposed to healthy adults (37.6%) (see Table 1). The sample was evenly distributed in regard to respondent years involved in the research. The majority of our sample (75.6%) reported that they received some to all of their research funding from federal sources. Our sample was overwhelmingly White (81.4%), which closely represents the demographic distribution of UW Madison faculty (White; 76%) and noninstructional academic staff (White; 79.8%) [15].

**Table 1.** Survey respondent characteristics

		*n* = 279	%
Primary school affiliation	School of Medicine and Public Health	228	81.4
	School of Nursing	10	3.6
	School of Education	6	2.1
	College of Letters and Science	5	1.8
	School of Pharmacy	4	1.4
	College of Agricultural and Life Sciences	4	1.4
	Other (engineering, veterinary medicine, environmental studies, etc.)	22	8.3
Research type (check all that apply)	Clinical	202	72.4
	Multisite trials	184	65.9
	Behavioral/Social Science	98	35.1
	Health Services Research	93	33.3
	Health Disparity Research	57	20.4
	Some/most all federal	211	75.6
	Some/most/all private industry	127	45.5
	Some/most/all foundation	97	34.8
	Some/most/all internal	121	43.4
	Other	34	12.2
Role in research (check all that apply)	PI	140	50.2
	Co-I	133	47.7
	Project Manager	69	24.7
	Recruiter or Outreach staff	74	26.5
	Clinical Trials Coordinator	85	30.5
	Regulatory/Compliance staff	62	22.2
	Other	24	8.6
Years in research	0–5 years	76	27.2
	6–10 years	60	21.5
	11–20 years	75	26.9
	More than 20 years	66	23.7
Recruits a lot or mostly	Healthy adults or children	105	37.6
	Adults or children with a specific health condition	203	72.8
Race/ethnicity (check all)	Latino or Hispanic	10	3.6
	American Indian or Alaska native	0	-
	Asian	26	9.3
	Black or African-American	11	3.9
	Native Hawaiian or Pacific Islander	0	-
	White	227	81.4
	Other	7	2.5

Overall, respondents had a high level of agreement about the value of diverse recruitment. The majority (87.1%) believed diversity to be very important/extremely important. However, when asked how much diversity was a priority in their specific research program, only 38.3% responded with “a lot” or “a great deal.” This difference was also revealed in the averages of Best Practices knowledge (BP knowledge) and Best Practices implementation (BP implementation) scales which were measured on a 1–5 scale with 5 as the highest. Average scores for these scales were 4.0, 2.9, respectively.

**Fig. 1.**
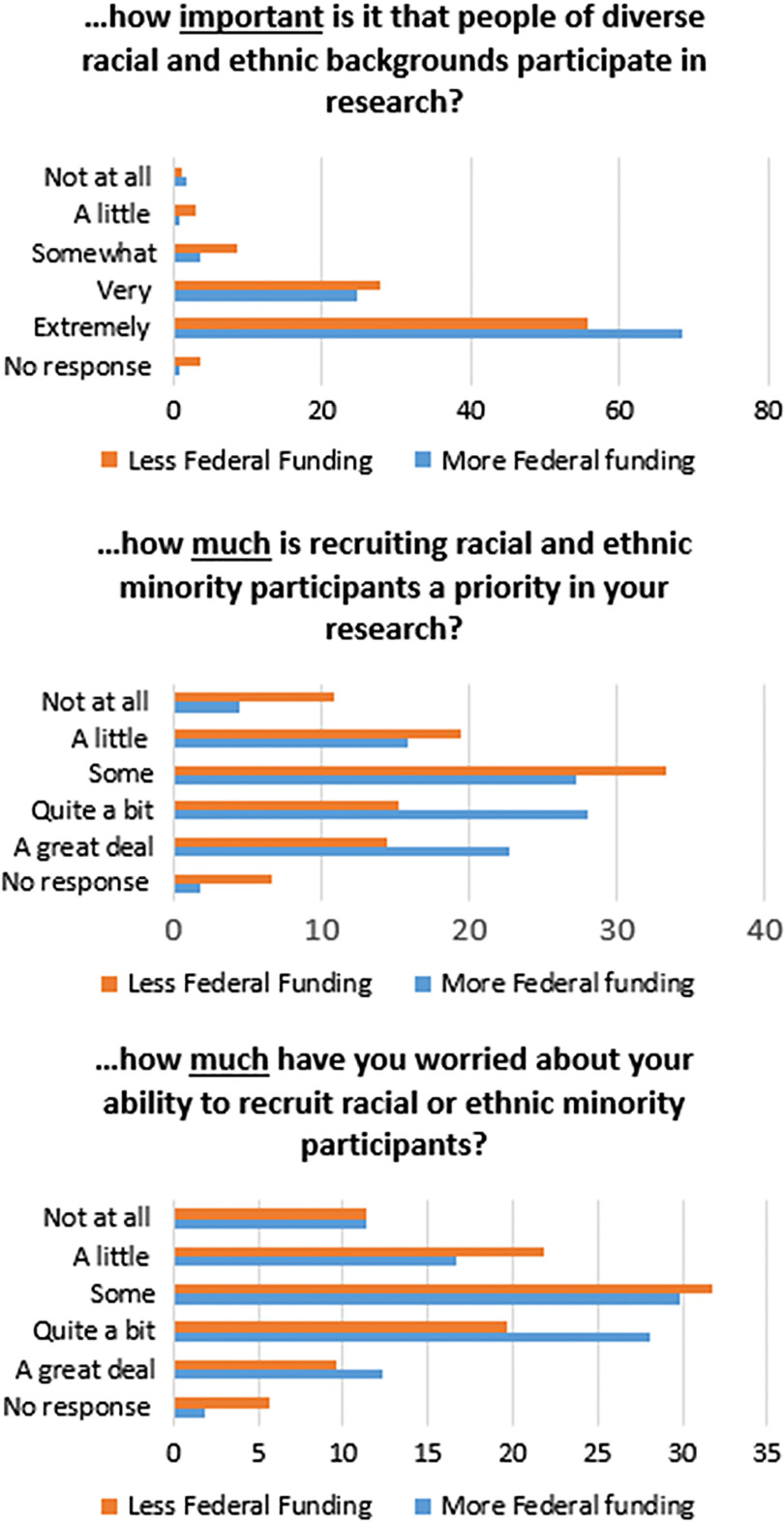
Comparison of respondents with high/low reported federal funding on relative “importance” of diversity in research samples.

Belief in the importance of diversity in research generally and in researchers’ specific work varied between those with high reported use of federal funding (e.g., NIH) and those with low use of federal funding. Mann–Whitney *U* tests were computed to examine these differences. There were statistically significant differences between high and low-funding levels on the following items: (1) How important is it that people of diverse racial and ethnic backgrounds participate in research? (*U* = 7858.5, *Z* = −2.052, *P* = .040); (2) How much is recruiting racial and ethnic minority participants a priority in your research? (*U* = 6736.5. *Z* = −3.139, *P* = .002); and (3) How much have you worried about your ability to recruit racial or ethnic minority participants? (*U* = 7014.5, *Z* = −2.522, *P* = .012) (see Fig. 1). Reporting of higher use of federal funding was also associated with higher scores on BP knowledge (4.1 vs. 3.8; *P*=.01) and, to a much larger degree, BP implementation (3.1 vs. 2.7; *P* < .001).

Other groups that scored highly on the BP knowledge scale were those recruiting mostly/all healthy participants and those with less years of experience in research. Those recruiting mostly/all healthy participants were also more likely to implement best practices as were those who reported receiving formal training in general (not URM specific) recruitment/retention practices (see Table 2).

**Table 2.** Comparisons of best practices (BP) knowledge and Implementation scales

Item		BP knowledge			BP implementation		
		Mean	SD	*P*	Mean	SD	*P*
Years in research	1–9 years	4.2	.71,671	<.001	2.9	.93,398	ns
	10+ years	3.8	.93,715		2.9	1.00631	
Participant type	Healthy participants	4.1	.76,174	<.01	3.2	.96,879	<.001
	Participants with specific diagnosis	3.8	.88,564		2.7	.92,764	
Had recruitment training	Training	4.0	.87,960	n/a	3.1	.93,402	<.001
	No training	4.0	.83,881		2.6	.98,384	

SD = standard deviation.

## Discussion

Overall, we identified a high level of abstract recognition of the importance of participant diversity that respondents did not generally see as impacting their individual work either in terms of prioritizing diversity or implementing best practices in URM engagement. There is some variation in our sample, however, that is encouraging. That individual newer to research (less than 10 years of experience) were found to have a greater knowledge of best practices may be an indication of an educational trend toward a greater emphasis placed on the value of diversity [1–3]. Similarly, we find that researchers and team members using more federal funding are significantly more likely than others to: (1) believe in the importance of research diversity generally and in their own research; (2) be knowledgeable about best practices; and (3) use best practices in their research. These data suggest that federal efforts to encourage inclusivity in research samples may be contributing to a positive trend [6]. However, the relatively low implementation of best practices across our sample suggests that, as others have found, efforts are inadequate [2, 3].

Although some respondents scored higher on the BP knowledge and implementation scales, our findings point to the existence of an overall gap between the belief in the importance of URM recruitment and the implementation of strategies to improve inclusion in research. Understanding the cause of this gap requires exploration with a larger, representative, perhaps, national sample. However, others have reported barriers caused by researcher and team member perceptions that URM is unwilling to participate, do not typically meet eligibility requirements, make unsuitable research participants, and are not among the clinic population from which samples are drawn [16,17]. The reported greater use of best practices for URM engagement among respondents working with healthy participants may reflect the ability to recruit outside of the clinic population.

Finally, the association with training and the reported implementation of best practices in URM recruitment are also encouraging although the percentage of researchers that receive specific training tends to be relatively low [16–18]. Trainings include the Building Trust Between Minorities and Researchers curriculum [19] and the Faster Together, Enhancing the Recruitment of Minorities in Clinical Trials program. Training programs report increases in self-confidence, communication skills, improved quality of informed consent and, for some, increases in recruitment numbers [20]. However, rigorous evaluations to include tracking of actual enrollment numbers have been limited to date [20].

Overall, our findings are mixed. We find evidence of a culture shift toward improving URM research engagement, perhaps stemming from NIH policy. However, researchers still need support in: (1) understanding that the goals of URM inclusion have implications for their specific work; and (2) translating strategies for URM inclusion into active recruitment and retention plans. These data call for education with less emphasis on abstract recruitment ideals and more on concrete ways to connect this knowledge to practice.

### Limitations

There are several limitations to this single-site study that impact generalizability. Our goal to guide the development of education programming specifically for researchers affiliated with UW-ICTR, ultimately, limited the reach of our findings. Readers should note that relative lack of diversity in our own sample, which reflects the distributions of race and ethnicity among researchers at our institution but is not representative of other institutions or the researcher workforce nationally. Another study limitation relates to the low response rate. While generally in line with expectations for web-based surveys among samples of health researchers/clinicians [12], this low rate limits generalizability. Finally, readers should note that the reported effect sizes, while significant, are relatively small. Nonetheless, we believe that this study provides an indication of an underlying problem in research inclusion that should be substantiated in further studies. Such studies should incorporate rigorous methods and nationally representative samples and be conducted across CTSA hubs or other national research infrastructure networks.

## Supplementary material

For supplementary material accompanying this paper visit https://doi.org/10.1017/cts.2020.554. click here to view supplementary material

## References

[1] Duma N , Vera Aguilera J , Paludo J , et al. Representation of minorities and women in oncology clinical trials: review of the past 14 years. Journal of Oncology Practice 2018; 14(1): e1–e10. 29099678 10.1200/JOP.2017.025288

[2] Knepper TC , McLeod HL . When will clinical trials finally reflect diversity? Nature 2018; 557(7704): 157–159. 29743700 10.1038/d41586-018-05049-5

[3] Oh SS , et al. Diversity in clinical and biomedical research: a promise yet to be fulfilled. PLoS Medicine 2015; 12(12): e1001918. 26671224 10.1371/journal.pmed.1001918 PMC4679830

[4] Shin J , Doraiswamy PM . Underrepresentation of African-Americans in Alzheimer’s trials: a call for affirmative action. Frontiers in Aging Neuroscience 2016; 8: 123. 27375473 10.3389/fnagi.2016.00123 PMC4891330

[5] Bhopal R . Ethical issues in health research on ethnic minority populations: focusing on inclusion and exclusion. Research Ethics 2008; 4(1): 15–19.

[6] Chen MS , Lara PN , Dang JHT , Paterniti DA , Kelly K . Twenty years post-nih revitalization act: renewing the case for enhancing minority participation in cancer clinical trials. Cancer 2014; 120(7): 1091–1096. 24643646 10.1002/cncr.28575 PMC3980490

[7] Food, Administration D. Enhancing the Diversity of Clinical Trial Populations—Eligibility Criteria, Enrollment Practices, and Trial Designs Guidance for Industry, 2019. (https://www.fda.gov/regulatory-information/search-fda-guidance-documents/enhancing-diversity-clinical-trial-populations-eligibility-criteria-enrollment-practices-and-trial)

[8] Heller C , Balls-Berry JE , Nery JD , et al. Strategies addressing barriers to clinical trial enrollment of underrepresented populations: a systematic review. Contemporary Clinical Trials 2014; 39(2): 169–182. 25131812 10.1016/j.cct.2014.08.004 PMC6936726

[9] Corbie-Smith G , Thomas SB , George DMMS . Distrust, race, and research. Archives of Internal Medicine 2002; 162(21): 2458–2463. 12437405 10.1001/archinte.162.21.2458

[10] Ahmed SM , Young SN , DeFino MC , Franco Z , Nelson DA . Towards a practical model for community engagement: advancing the art and science in academic health centers. Journal of Clinical and Translational Science 2017; 1(5): 310–315. 29707251 10.1017/cts.2017.304 PMC5915810

[11] Sussman AL , Cordova C , Burge MR . A comprehensive approach to community recruitment for clinical and translational research. Journal of Clinical and Translational Science 2018; 2(4): 249–252. 30775020 10.1017/cts.2018.324 PMC6374319

[12] Quinn SC , Butler III J , Fryer CS , et al. Attributes of researchers and their strategies to recruit minority populations: results of a national survey. Contemporary Clinical Trials 2012; 33(6): 1231–1237. 22771575 10.1016/j.cct.2012.06.011 PMC3468713

[13] Tanner A , Kim S-H , Friedman DB , Foster C , Bergeron CD . Promoting clinical research to medically underserved communities: current practices and perceptions about clinical trial recruiting strategies. Contemporary Clinical Trials 2015; 41: 39–44. 25542611 10.1016/j.cct.2014.12.010

[14] Sullivan GM , Artino AR . Analyzing and interpreting data from likert-type scales. Journal of Graduate Medical Education 2013; 5(4): 541–542. 24454995 10.4300/JGME-5-4-18 PMC3886444

[15] Office of Policy Analysis & Research. University of Wisconsin System Accountabilty Dashboard: Faculty & Staff. Accountability Dashboard, 2017. (https://www.wisconsin.edu/accountability/faculty-and-staff/).

[16] Niranjan SJ , Martin MY , Fouad MN , et al. Bias and stereotyping among research and clinical professionals: Perspectives on minority recruitment for oncology clinical trials. Journal of Clinical Oncology 2019; 37(27): 152–152. 10.1002/cncr.32755 32147815

[17] Durant RW , Wenzel JA , Scarinci IC , et al. Perspectives on barriers and facilitators to minority recruitment for clinical trials among cancer center leaders, investigators, research staff and referring clinicians. Cancer 2014; 120(7): 1097–1105. 24643647 10.1002/cncr.28574 PMC4395557

[18] Niranjan SJ , Durant RW , Wenzel JA , et al. Training needs of clinical and research professionals to optimize minority recruitment and retention in cancer clinical trials. *Journal of Cancer Education* 2019; 34: 26–34. 10.1007/s13187-017-1261-0 PMC5797508 28776305

[19] Hartnett T . Minority research: building trust project aims to improve participation in strengthen capacity for investigators and IRBs. Research Practice 2011; 12(5): 157.

[20] Delaney H , Devane D , Hunter A , et al. Limited evidence exists on the effectiveness of education and training interventions on trial recruitment; a systematic review. Journal of Clinical Epidemiology 2019; 113: 75–82. 31128220 10.1016/j.jclinepi.2019.05.013

