# Supplementary material for: Training needs of investigators and research team members to improve inclusivity in clinical and translational research participation
Source: J Clin Transl Sci. 2020 Nov 5;5(1):e57. doi: 10.1017/cts.2020.554 (PMC8057444; doi:10.1017/cts.2020.554)
Supplement: Supplementary file 1 [file S2059866120005543sup001.pdf]

Training Needs of Investigators and Research Teams Survey

1. What is your primary school & department/unit affiliation? ______________

2. In the last 3 years, have you been involved in the following kinds of human subject research?

	Yes	No
Behavioral or social science	○	○
Clinical trials	○	○
Health services research	○	○
Intervention studies	○	○
Health disparity/equity research	○	○
Multi-site studies	○	○
Observational studies	○	○
Other human subject research? Please tell us:	○	○

3. Still thinking about the research projects you have been involved with in the last 3 years, about how much financial support was supplied by the following funders?

	None	A little	Most	Some	All
Private industry	○	○	○	○	○
Federal funding such as NIH or NSF	○	○	○	○	○
Foundations	○	○	○	○	○
Internal UW funding (ex: ITCR pilot or other seed program	○	○	○	○	○
Other funder, please tell us:	○	○	○	○	○

4. In the last 3 years, about what percentage of your work effort has been devoted to the implementation of human subjects research? (rough estimate is fine)

○ 25% or less

○ 26% to 50%

○ 51% to 75%

○ 76% or more

5. In the last 3 years, how much of your role in research involved…

	None	A little	Most	Some	All
…front line recruitment or retention (i.e. personally interacting with potential or existing research participants?	○	○	○	○	○
… the development of recruitment or retention plans?	○	○	○	○	○

6. In the last 3 years, which of the following roles have you had …

	Yes	No
Principal investigator or PI	○	○
Co-investigator or Co-I	○	○
Project manager	○	○
Recruiter or outreach staff	○	○
Clinical trial coordinator	○	○
Regulatory/compliance staff	○	○
Other study staff? Please tell us:	○	○

7. Thinking about the research study(ies) you worked in most recently, in general, <u>how much</u> do the Principal Investigator (s) you have worked with…

	Not at all	A little	Some	Quite a bit	A great deal
…respect your opinions regarding study recruitment?	○	○	○	○	○
…include you in the planning of the study recruitment or retention plans?	○	○	○	○	○
…include you in decisions regarding the study recruitment plan after enrollment had begun?	○	○	○	○	○

8. Still thinking about the research study(ies) you worked in most recently, in general, <u>how realistic</u> were the Principal Investigator's expectations about the number and types of participants that could be recruited into the study?

○ Not at all

○ A little

○ Somewhat

○ Very

○ Extremely

9. Have you had <u>cultural competence or diversity training</u> opportunities in your training (ex: in school) or while you have been working?

○ Yes

○ No

10. Have you had research participant recruitment or retention training opportunities in your training (ex: in school) or while you have been working?

○ Yes

○ No

11. In the last 3 years, how much have you or your team focused on recruiting research participants from the following populations?

	**Not at all**	**A little**	**Some**	**Quite a bit**	**A great deal**
Healthy adults or children	○	○	○	○	○
Adults or children with a specific health condition	○	○	○	○	○
Students	○	○	○	○	○
Other population, please tell us:	○	○	○	○	○

12. Which one of the following best describes how your team has recruited research participants in the last 3 years? Have you recruited…

○ …mostly through the health care system, such as physical referrals or clinic patients

○ …mostly outside the health care system, such as through social media, public ads or events.

○ …more or less equally through the health care system and outside the health care system

○ …from other settings? Please tell us:

13. Thinking about human subjects research GENERALLY (your specific research experience may vary)…

	**Not at all**	**A little**	**Somewhat**	**Very**	**Extremely**
…how important is having community based recruitment or retention strategies (ex: having community partnerships, community advisory board, etc)?	○	○	○	○	○
…how important is it that people of diverse racial and ethnic backgrounds participate in research?	○	○	○	○	○
...how important is it that the research team be made up of people of diverse racial or ethnic backgrounds?	○	○	○	○	○

14. Still thinking about human subjects research GENERALLY but now focusing on the recruitment of RACIAL AND ETHNIC MINORITY PARTICIPANTS

	**Not at all**	**A little**	**Somewhat**	**Very**	**Extremely**
…how important is having community based recruitment or retention strategies (ex: having community partnerships, community advisory board, etc.)?	○	○	○	○	○
...how important is it to hire recruitment /retention staff of the same race or ethnicity as the people you hope to enroll?	○	○	○	○	○
…how important is it to plan extra time to successfully recruit participants?	○	○	○	○	○
…how important is it to develop tailored recruitment or retention materials?	○	○	○	○	○

15. Still thinking GENERALLY about research, how significant are the following BARRIERS to recruiting or retaining participants from RACIAL OR ETHNIC MINORITY BACKGROUNDS...

	**Not at all**	**A little**	**Somewhat**	**Very**	**Extremely**
Potential participants' distrust due to past research abuse (like the Tuskegee Syphilis study)	○	○	○	○	○
Potential participants' negative perceptions of research, such as fear of being treated "like a guinea pig"	○	○	○	○	○
Potential participants' low literacy or low health literacy	○	○	○	○	○
Language barriers	○	○	○	○	○
University or hospital policy or procedures (ex: distribution of incentives; HIPPA requirements; IRB requirements)	○	○	○	○	○
Study specific protocols/procedures	○	○	○	○	○
Attitudes or behaviors of members of the research team	○	○	○	○	○

16. Now thinking about YOUR WORK involving human subjects research, SPECIFICALLY …

	**Not at all**	**A little**	**Somewhat**	**Very**	**Extremely**
…how knowledgeable do others consider you to be about research participant recruitment?	○	○	○	○	○
…how knowledgeable do others consider you to be about research participant retention?	○	○	○	○	○
…how much is recruiting racial and ethnic minority participants a priority in your research?	○	○	○	○	○
…how successful have you or your team(s) been in recruiting racial and ethnic minority participants to your research studies?	○	○	○	○	○
…how much have you worried about your ability to recruit racial or ethnic minority participants?	○	○	○	○	○

17. Still thinking about YOUR WORK involving human subjects research, SPECIFICALLY…

	**Not at all**	**A little**	**Somewhat**	**Very**	**Extremely**
...how much have you/your team(s) made an effort to hire diverse recruitment staff?	○	○	○	○	○
…how much have you/your team(s) incorporated extra time to successfully recruit participants?	○	○	○	○	○
…how much have you/your team(s) used tailored recruitment materials for specific populations?	○	○	○	○	○
…how much have your project(s) used community based recruitment strategies (ex: having community partnerships or a community advisory board)?	○	○	○	○	○
...how easy have you found it to be to get recruitment and study materials translated into the languages needed?	○	○	○	○	○

18. In your work in human subjects research, how often have you seen conflicts between members of research study teams due to racial or ethnic difference?

- ◯ Never
- ◯ Rarely
- ◯ Sometimes
- ◯ Very often
- ◯ Extremely often

19. About how many years have you been involved in human subjects research?

- ◯ 0 to 5 years
- ◯ 6 to 10 years
- ◯ 11 to 20 years
- ◯ More than 20 years

20. Which of the following credentials do you have? (Please check all that apply)

- ◯ Medical Doctor or Doctor of Osteopathic Medicine
- ◯ Doctor of Philosophy
- ◯ Doctor of Pharmacy
- ◯ Registered Nurse
- ◯ Master of Public Health
- ◯ Master of Arts or Master of Science
- ◯ Bachelor of Art or Bachelor of Science Master of Public Health
- ◯ Certified Clinical Research Coordinator

○ Other credential: ________________

21. Are you Hispanic or Latino?

○ Yes

○ No

22. Which of the following describe your race? (Please check all that apply)

○ American Indian or Alaska Native

○ Asian

○ Black or African American

○ Native Hawaiian or Pacific Islander

○ White

○ Other? Please tell us:

23. What is your gender?

○ Man

○ Nonbinary

○ Woman

○ Not listed? Please tell us: ____
